# Adjusted delivery, stronger outcomes: Selinexor therapeutic adaptation and positioning in previously treated multiple myeloma

**DOI:** 10.1111/bjh.70711

**Published:** 2026-07-21

**Authors:** Muhamed Baljevic

**Affiliations:** ^1^ Department of Internal Medicine Vanderbilt University Medical Center Nashville Tennessee USA; ^2^ Vanderbilt‐Ingram Cancer Center Vanderbilt University Medical Center Nashville Tennessee USA

**Keywords:** dosing, myeloma, selinexor, sequencing

## Abstract

In patients with lenalidomide‐refractory multiple myeloma from the BOSTON trial subgroup analysis,^1^ selinexor (SEL) dose reduction versus full dosing substantially improved efficacy outcomes, including higher overall response rate (74.3% vs. 55.6%), deeper responses (≥ very good partial response: 48.6% vs. 11.1%), longer duration of response (15.3 vs. 4.2 months), longer time to next treatment (14.8 vs. 4.8 months) and longer progression‐free survival (13.9 months vs. 5.1 months). Selinexor dose reduction further improved tolerability, enabling longer treatment duration, with non‐haematological adverse events such as nausea, fatigue, diarrhoea, decreased appetite and vomiting all decreased meaningfully after the first SEL dose reduction. Quality of life improved meaningfully also: global health status quality assessed via European Organization for Research and Treatment of Cancer Quality of Life Questionnaire improved more than twice as much in the SEL‐reduced group versus the full‐dose group, with the dose‐reduced patients exceeding the clinically meaningful 10‐point improvement threshold after dose reduction.

Commentary on: Delimpasi et al. Benefit of selinexor dose reduction on outcomes with selinexor, bortezomib and dexamethasone in patients with lenalidomide‐refractory multiple myeloma: Subgroup analysis of the BOSTON trial. Br J Haematol 2026; 208:2260‐2264.

In the recent issue, Delimpasi et al. report on the benefit of Selinexor (SEL) dose reduction on the outcomes with SEL, bortezomib and dexamethasone in patients with lenalidomide‐refractory multiple myeloma (MM) from the Bortezomib Once‐weekly Selinexor Treatment Or Novel (BOSTON) trial subgroup analysis.^1^ Their work highlights several key observations: (a) SEL dose reduction substantially improved efficacy outcomes: patients with a SEL dose reduction achieved a much higher overall response rate (ORR) (74.3% vs. 55.6%), deeper responses [≥very good partial response (VGPR): 48.6% vs. 11.1%], longer duration of response (15.3 vs. 4.2 months) and longer time to next treatment (14.8 vs. 4.8 months). Median progression‐free survival (PFS) after comparable duration of median follow‐up (27–28 months) was 13.9 months in the SEL‐reduced group versus only 5.1 months in the full‐dose group—a striking difference even with SEL‐dose reduced group harbouring higher proportion of high‐risk cytogenetics (60% vs. 44%, respectively); (b) SEL dose reduction improved tolerability, enabling longer treatment duration: non‐haematological adverse events such as nausea, fatigue, diarrhoea, decreased appetite and vomiting all decreased meaningfully after the first SEL dose reduction. This improved tolerability in turn likely facilitated longer exposure via three times longer period on (rather than off) therapy in patients in the SEL‐dose reduction, which supported improved outcomes; (c) quality of life improved meaningfully with dose reduction: global health status quality assessed via European Organization for Research and Treatment of Cancer Quality of Life Questionnaire improved more than twice as much in the SEL‐reduced group versus the full‐dose group, with the dose‐reduced patients exceeding the clinically meaningful 10‐point improvement threshold after dose reduction. This reinforces the clinical notion that reducing the SEL dose is not a treatment failure, but a patient‐oriented optimization that supports both efficacy, tolerability and overall well‐being.

Even as observations by Delimpasi et al. reinforce the prior comprehensive patient‐level data analysis from the BOSTON, Selinexor and Backbone Treatments of Multiple Myeloma Patients (STOMP), Selinexor Treatment of Refractory Myeloma (STORM) and Exportin‐1 (XPO1) Oral Therapeutics (XPORT)‐MM‐028 clinical trials, into relationships between SEL dosing schedule, regimen toxicities and efficacy,^2^ the question remains: how do SEL‐containing regimens best fit into an evolving MM treatment paradigm of heavily pre‐treated patients? In particular, how can we leverage SEL regimens further to assist in the management of rapidly evolving group of multiclass refractory patients, including those who progress after B‐cell maturation antigen (BCMA)‐ and/or G protein‐coupled receptor class C group 5 member D (GPRC5D)‐treatments. This emerging group of patients that have disease resistant to T‐cell engaging therapies represent a unique subset of patients that with high unmet need, uncertain treatment options and overall dismal prognosis.

Selinexor reversibly inhibits nuclear export of tumour suppressor proteins (TSPs), growth regulators and mRNAs of oncogenic proteins by blocking the XPO1. Nuclear accumulation of TSPs and abrogation of oncogenic messenger ribonucleic acid translation lead to reductions in cell cycle arrest and apoptosis of cancer cells. Additionally, nuclear retention of proteins like inhibitor of nuclear factor kappa B alpha, signal transducer and activator of transcription 1/3, forkhead box O 1/3, retinoblastoma and the glucocorticoid receptor also remodels the inflammatory and immune microenvironment while complementing cellular immunotherapy approaches through (Figure 1). This in turn leads to suppression of pro‐inflammatory gene transcription, modulation of T‐cell activation and exhaustion, increased immunogenicity in tumour cells, less inhibition of immune effector cells and potential enhancement of immune‐mediated killing.^3^

**FIGURE 1:**
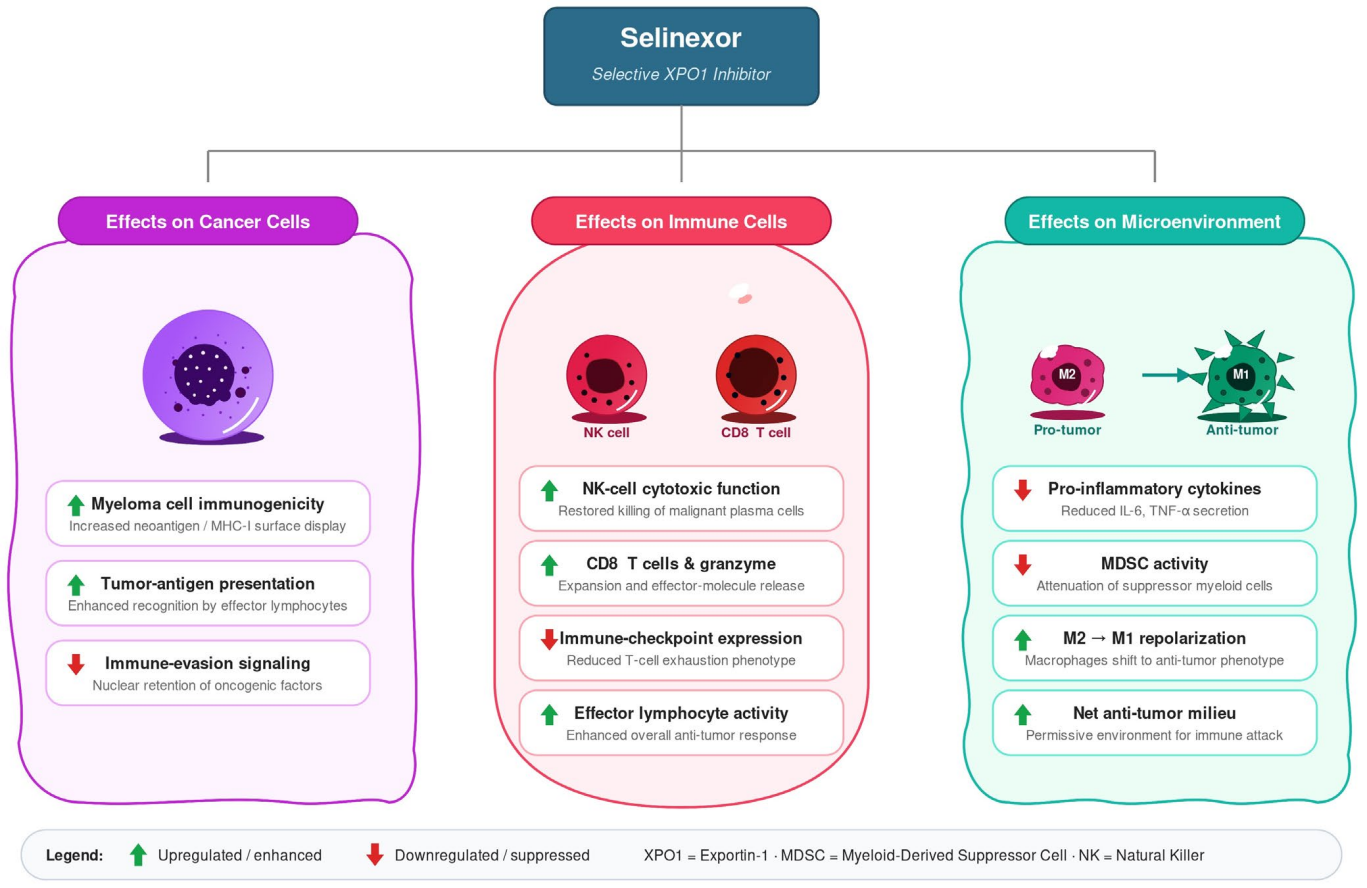
Selinexor influences malignant cells, immune effector cells and tumour/immune microenvironment.

Clinical outcomes and resistance to various forms of T‐cell redirecting therapies (TCRTs) are heavily influenced by T‐cell exhaustion, antigen loss and an immunosuppressive tumour microenvironment. Accumulating preclinical and translational data are suggesting that immunomodulatory effects of SEL therapy may suppress the emergence of resistance pathways, which could limit the TCRT efficacy and durability. Evidence of SEL clinical activity peri‐TCRT was seen in several studies that include: (a) SEL as a bridging therapy before idecabtagene vicleucel was associated with PFS benefit of 9.8 months, comparable to that of immunomodulatory drug‐based bridging^4^; (b) in a heavily pre‐treated relapsed/refractory MM (RRMM), SEL‐containing regimens before BCMA chimeric antigen receptor (CAR) T‐cell therapy were associated with median post–CAR‐T PFS of 8 months and an overall survival (OS) of 35.9 months^5^; (c) in a retrospective study on 7 RRMM patients, SEL‐based treatments immediately prior to CAR‐T‐cell apheresis resulted in 100% ORR, with all patients achieving ≥VGPR after CAR‐T treatment^6^; (d) the combination of two‐step SEL dosing and BCMA CAR‐T, CT103A, has shown preliminary evidence of activity in extra‐medullary multiple myeloma^7^; weekly SEL in combination with pomalidomide or carfilzomib in particular showed efficacy in difficult‐to‐treat, multi‐class RRMM, including that refractory to prior BCMA‐directed therapies.^8^

Despite the fact that contemporary treatment advances have raised the possibility of long‐term remission and functional cure in at least the subset of patients with previously heavily pre‐treated disease,^9^ MM is largely still incurable, with a third of patients dying within the first year of diagnosis (patients aged 75 years or older representing the highest proportion of deaths).^10^ The long‐term success in multi‐class RRMM, which has one of the highest unmet needs in the field, remains particularly elusive. There is therefore an increasing need to both optimize the therapeutic sequencing among the available and up‐and‐coming TCRTs and also tackle the resistance to TCRTs via strategic interspersing of other mechanistic anti‐MM drug classes, which possess the capacity to possibly lessen T‐cell exhaustion and augment the performance of immune therapies whose potency is tightly connected to T‐cell fitness and other forms of immune competence. Table 1 shows some of the ongoing trials using SEL in the context of T‐cell redirecting therapies that have either already been started or are in preparation for activation (personal communication).

**TABLE 1:** Current/planned studies combining selinexor with T‐cell redirecting therapies.

Study title	Principal investigator & organization	Status	Patient population
A Phase 2B study of selinexor (KPT‐330), in combination with carfilzomib, daratumumab, pomalidomide or elranatamab in patients with multiple myeloma relapsing on current therapy	Dr. Noa Biran Hackensack University (NCT04661137)	Enrolling	Patients must be actively refractory to or progressing while on an elranatamab‐containing regimen
A Study of a Fully Human BCMA‐targeting CAR (CT103A) Combined With Selinexor in Patients With Relapsed/Refractory Extramedullary Multiple Myeloma	Dr. Chunrui Li Tongji Hospital, Tongji Medical College, Huazhong University of Science and Technology (NCT05201118)	Enrolling	A single‐centre, open Phase I study, to observe the effectiveness and safety of CT103A combined with different doses of selinexor in patients with relapsed/refractory extra‐medullary multiple myeloma
A phase II safety and efficacy study of selinexor in combination with bispecific antibody in patients with relapsed/refractory multiple myeloma	Dr. Cristiana Chase Duke University (NCT06822972)	Enrolling	Patients with RRMM who are receiving commercial bispecific antibody treatment. Must have received at least 4 prior lines of therapy, including a proteasome inhibitor, an immunomodulatory agent and a cluster of differentiation (CD)38 monoclonal antibody.
CAROUSEL study—using selinexor in conjunction with CAR‐T to deepen response in relapsed refractory multiple myeloma	Dr. Edward Faber University of Cincinnati *Planned*	Planning	Patients with high‐risk and ultra‐high‐risk RRMM undergoing CAR‐T therapy. *High/ultra‐high risk defined as: Presence of at least one high‐risk cytogenetic abnormality* (e.g. *del*(*17p*), *t*(*4;14*)), *extra‐medullary disease, primary refractory disease within 12 months of first‐line treatment or penta‐refractory disease*
Selinexor maintenance post CAR‐T cell therapy for Multiple Myeloma	Dr. Mark Schroeder Washington University *Planned*	Planning	Patients with triple‐class exposed or refractory MM who have received cilta‐cel and have a high‐risk of relapse. *High‐risk defined as: Adverse risk cytogenetics, extra‐medullary disease or less than a complete response* (*CR*) *at Day 100 post‐CAR‐T*.

Selinexor is an approved but underutilized drug in RRMM that can be highly effective when managed properly, as shown by Delimpasi et al. Unfortunately, despite the abundance of powerful TCRTs, the best treatment outcomes associated with long‐term success are still not experienced by the majority of patients, particularly those triple‐class/BCMA/GPRC5D refractory. Optimal dosing of SEL with intentional and strategic sequencing—such as ‘sandwiching’ of SEL‐containing regimens before and after TCRTs—is one of the approaches that carries significant potential with SEL and we look forward to witnessing the results it may yield.

## AUTHOR CONTRIBUTIONS

**Muhamed Baljevic:** Conceptualization; writing – original draft; formal analysis.

## FUNDING INFORMATION

No funding was provided for this editorial.

## CONFLICT OF INTEREST STATEMENT

Muhamed Baljevic reports consultancy for AbbVie, J&J, Sanofi‐Genzyme; advisory board participation for Janssen Biotech, J&J, Sanofi‐Genzyme, Pfizer, Prothena, AbbVie, Legend Biotech; research grant support from Pfizer and J&J; data safety monitoring board member for Delta‐Fly; independent review committee for Parexel International/GSK, AbbVie.

## Data Availability

Data are available upon request.
